# Supplementary material for: Sample preparation and data collection for serial block face scanning electron microscopy of mammalian cell monolayers
Source: PLoS One. 2024 Aug 9;19(8):e0301284. doi: 10.1371/journal.pone.0301284 (PMC11315281; doi:10.1371/journal.pone.0301284)
Supplement: S1 File — (PDF) [file pone.0301284.s001.pdf]

Feb 02, 2024 Version 2

Sample preparation and data collection for Serial Block Face Scanning Electron Microscopy of Mammalian Cell Monolayers V.2

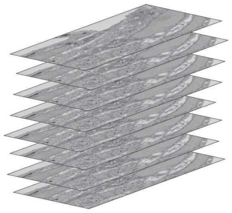

DOI
dx.doi.org/10.17504/protocols.io.e6nvwdz5zlmk/v2

Noelle Antao[1], Joseph Sall[2], Christopher Petzold[2], Damian C. Ekiert[1,3], Gira Bhabha[1], Feng-Xia Liang[2]

[1]Department of Cell Biology, New York University School of Medicine, New York, NY, USA;
[2]Office of Science and Research Microscopy Laboratory, New York University School of Medicine, New York, NY, USA;
[3]Department of Microbiology, New York University School of Medicine, New York, NY, USA

Noelle Antao

OPEN ACCESS

DOI: dx.doi.org/10.17504/protocols.io.e6nvwdz5zlmk/v2

Protocol Citation: Noelle Antao, Joseph Sall, Christopher Petzold, Damian C. Ekiert, Gira Bhabha, Feng-Xia Liang 2024. Sample preparation and data collection for Serial Block Face Scanning Electron Microscopy of Mammalian Cell Monolayers. **protocols.io** https://dx.doi.org/10.17504/protocols.io.e6nvwdz5zlmk/v2Version created by Noelle Antao

License: This is an open access protocol distributed under the terms of the **Creative Commons Attribution License**, which permits unrestricted use, distribution, and reproduction in any medium, provided the original author and source are credited

Protocol status: Working
We use this protocol and it's working

Created: February 01, 2024

Last Modified: February 02, 2024

Protocol Integer ID: 94576

Funders Acknowledgement:
NYU Microscopy Core (partial funding)
Grant ID: NIH/NCI P30CA016087

Abstract

Serial block face scanning electron microscopy (SBF-SEM) is a volume EM technique used to study biological samples including tissue and cellular samples. Here we describe detailed protocols for two sample embedding methods for in vitro tissue culture cells intended to be studied using SBF-SEM. The first protocol focuses on cell pellet embedding and the second on *en face* embedding. *En face* embedding can be combined with light microscopy, and this CLEM workflow can be used to identify specific biological events in a light microscope, which can then be imaged using SBF-SEM. We systematically outline the steps necessary to fix, stain, embed and image adherent tissue culture cell monolayers by SBF-SEM. Users experienced with electron microscopy sample preparation methodology will be able to complete this protocol in 10-11 days from initial seeding of cells in tissue culture to image acquisition.

Attachments

PDF
Table 1.pdf
30KB

PDF
TROUBLESHOOTING TABL...
27KB

PDF
Figure 1_protocols.i...
1.6MB

Image Attribution

Guidelines

TIMING

This protocol can be performed over multiple days. Once samples are embedded in resin they are stable at room temperature indefinitely once infiltrated by and polymerized in resin.

Step 1: Culturing mammalian cells, plus an additional 24 - 48 h for cells to grow

Step 2: Brightfield or fluorescence microscopy for CLEM workflow with *en face* samples, 1 - 2 h

Steps 3-5: 2 h fixation at room temperature plus an additional 24 h at 4 °C.

Step 6-19: Sample staining, embedding and polymerization, 5 days plus any additional time for reagent setup and clean up.

Step 20-28: Cell pellet sample trimming and mounting, 2 h plus any additional time for epoxy polymerization, reagent setup and clean up.

Steps 29-47: *en face* sample trimming and mounting, 4 h plus any additional time for epoxy polymerization, reagent setup and clean up.

Steps 48-49: sample loading on the SBF-SEM, 1 h plus an additional 12 h to let the system equilibrate.

Steps 50-62: sample imaging, several hours to days depending on the number of ROIs being collected, the volume assigned as well as pixel size and dwell time.

Materials

Biological materials

In this protocol we use Vero cells (African green monkey epithelial) infected with Encephalitozoon intestinalis, a microsporidian parasite **[6]**.

Reagents

- Dulbecco's Modified Eagle Medium, high glucose (DMEM:HG) (ThermoFisher Scientific, Cat no. 11995065)
- Dulbecco's phosphate-buffered saline (DPBS) (ThermoFisher Scientific, Cat no. 14190144)
- Trypsin-EDTA (ThermoFisher Scientific, Cat no. MT25053CI)
- Trypan Blue (ThermoFisher Scientific, Cat no.T10282)
- Fetal Bovine Serum (VWR 89510-188)
- 100X Non essential amino acids (Fisher Scientific, Cat no. 11-140-050)
- Milli Q water
- 16% paraformaldehyde (Electron Microscopy Sciences, EMS, Cat no. 15700)
- 25% glutaraldehyde (Electron Microscopy Sciences, Cat no. 16019)
- 0.4 M sodium cacodylate buffer, pH 7.2 (Electron Microscopy Sciences, Cat no. 11654)
- 4% Osmium Tetroxide (Electron Microscopy Sciences, Cat no. 19150)
- Potassium Ferrocyanide (same as potassium hexacyanoferrate (II) trihydrate, Sigma, Cat no. 60279)
- Thiocarbohydrazide (TCH, Electron Microscopy Sciences, Cat no. 21900)
- L-aspartic acid (Sigma-Aldrich, Cat no. A9256)
- Lead nitrate (Ted Pella Inc., Cat no. 19321)
- Ethanol, 200 proof (Electron Microscopy Sciences, Cat no. 15056)
- Durcupan ACM Epoxy (Electron Microscopy Science, Cat no. 14040)
- EMbed 812 Embedding Kit (Electron Microscopy Science, Cat no. 14121)
- Silver conductive epoxy (Chemtronics, Cat no. CW2400 Epoxy)

Equipment

- Biosafety cabinet
- CO_2 incubator (Heracell CO2 Incubators, ThermoFisher Scientific)
- Inverted light microscope to check confluence of cells (Carl Zeiss Microscopy LLC)
- Countess II Cell Counter (ThermoFisher Scientific)
- Invitrogen Countess Cell Counting Chamber Slides (ThermoFisher Scientific, Cat no. C10312)
- Ibitreat, 35 mm tissue culture treated dish (Ibidi, Cat no. 81156)
- 35 mm tissue culture dish; No. 1.5 Gridded Coverslip; 14 mm Glass Diameter (MatTek Corporation, Cat no. P35G-1.5-14-CGRD)
- Cell scraper (VWR 10062-904)
- Wood Applicator Sticks (Solon, Cat no. FBHOO360)
- Embedding mold (PELCO, Cat no. 10535)
- Pipette and pipette tips
- Chemical hoods
- Razor (EMS Cat# 72000-WA)
- Formar Support Copper Slot Grid (Electron Microscopy Science, Cat no. FF2010)

Specific equipment

- 3View SEM pin stub: 1.4 mm flat, 2 mm pin, 12 mm height (EMS catalog# 75959-02)

- 3View SEM pin stub: 2.4 mm flat, 2 mm Pin, 12.5 mm height (EMS Cat# 75959-03)
- Diatome Histo Diamond Knife (Diatome, Cat no. DH4540)
- Leica EM UC6 Ultramicrotome (Leica microsystems)
- Zeiss Gemini300 Field Emission Scanning Electron Microscopy with Gatan 3View (Carl Zeiss Microscopy GmbH) and a second generation FCC or relevant volume electron microscope

Software

- DigitalMicrograph (Gatan)
- Fiji

Reagent setup

- Complete DMEM: HG media containing 10% fetal bovine serum and 1X non essential amino acids.
- Complete DMEM: HG media containing 3% fetal bovine serum and 1X non essential amino acids.
- Fixation solution: 2.5% Glutaraldehyde, 2% paraformaldehyde in 0.1M Sodium Cacodylate Buffer (pH 7.2-7.4). The osmolarity of the original Karnovsky mixture (4% PFA and 5% glutaraldehyde) is too high (2010 mOsmol), therefore, half strength (2% PFA and 2.5% glutaraldehyde) of the aldehyde mixture is more popular today.
- TCH solution: 0.1g TCH to 10 mL ddH2O and place in a 60 °C oven for 1 h, agitate by swirling gently every 10 mins to facilitate dissolving. Filter solution through a 0.22 µm filter right before use.
- 2% Osmium in ddH_2O
- 1% Uranyl acetate in ddH_2O
- 1.5% potassium ferrocyanide in ddH_2O
- Aspartic acid stock solution: dissolve 0.998 g of L-aspartic acid in 250 mL ddH_2O water. Note: aspartic acid dissolves more readily at pH 3.8. The solution is stable for 1-2 months at 4 °C.
- Walton's lead aspartate solution**[1]**: dissolve 0.066g lead nitrate in 10 mL of the aspartic acid stock solution. Adjust the pH of the solution to 5.5 with 1M KOH.
- Durcupan ACM: Epoxy resin/ A (11.4 g); 964 hardener/ B (10 g); 964 accelerator/ C (0.3 g); Dibutyl phthalate / D (0.05-0.1 g) for en face samples
- Hard Epon: EMbed**[2]** 812 (5 mL); DDSA (2.25 mL); NMA (3 mL); BDMA (0.3 mL) for cell pellet samples.

Safety warnings

Caution steps in the protocol have been indicated. Please refer to the material safety data sheet of the compound to ensure its careful handling.

Culturing mammalian cells and sample fixation

1 Culture adherent mammalian cells for 24 - 48 h in a 60 mm or 100 mm tissue culture dish for cell pellet samples, or 35 mm tissue culture dish with a gridded glass bottom for CLEM samples (final density 3-6 x 10^5 cells) according to the standard protocol for the cell line. In our experiments, Vero cells were cultured in complete DMEM:HG media.

1.1 Note: For cell pellet samples, the cells can be collected when they reach 90% confluence. This ensures that there are enough cells to form a large cell pellet. For CLEM, the cells should be at 70% confluence or less, as this will allow you to track the position of a cell of interest by both light and electron microscopy.

2 For *en face* embedding of cells, proceed with light microscopy to identify specific cells of interest. Depending on the biological event being studied, you can use brightfield or fluorescence microscopy to identify cells of interest (Figure 1A). For cell pellet samples, proceed directly to step 4.

2.1 Critical step: 1) Image cells in an environmental chamber where temperature, humidity and CO_2 concentrations are maintained 2) Capture images at high magnification (40X or 60X) and low magnification (5X or 10X) of multiple regions of interest using DIC and fluorescence imaging, as appropriate, to identify cells of interest. Images collected at high magnification will allow you to record both the cell shape and a more precise position relative to the surrounding cells. From images collected at low magnification, a map of the cell location showing the gridded lines, numbers or letters can be used as a marker when trimming the EM sample block, and searching the target cell of interest under EM. We recommend selecting 2 - 4 regions of interest per sample, that are located far apart from each other on the dish. This ensures that each region of interest will be reliably recovered during the subsequent processing steps when the sample is physically cut away from the polymerized resin.

3 Discard tissue culture media, and gently pipette 2 mL of fixative (2% PFA and 2.5% glutaraldehyde in 0.1 M sodium cacodylate buffer (pH 7.2)) to fix the cells. For cell lines that are more sensitive, add 1 mL of pre-warmed 2x fixative (4% PFA and 5% glutaraldehyde in 0.2M sodium cacodylate buffer pH 7.2) directly into the dish with 1 mL of culture media.

3.1 Critical step: fixative solution should be freshly prepared

3.2 Note: the cells can be fixed first before light microscopy imaging. The fixative can be replaced with PBS, and imaging can be done under the light microscope without an environmental chamber.

4 For cell pellet sample fixation, after 1 min of fixation with 2% paraformaldehyde and 2.5% glutaraldehyde in 0.1 M sodium cacodylate buffer (pH 7.2), detach cells from the cell culture

dish using a cell scraper. Transfer the cells into a 1.5 mL tube, and centrifuge immediately at 2400 *xg* for 2 min. Rotate the tube 180° and centrifuge at 2400 *xg* for an additional 2 min. Cells should be observed as a pellet at the bottom of the tube.

4.1 Critical step: 1) Scrape cells in a single direction, such that you observe a sheet of cells coming off the dish. 2) In-dish fixation should be limited to less than 3 mins to avoid disintegration of the cell monolayer which can make recovery of the dense cell pellet during centrifugation tough. 3) For cell pellet samples, avoid centrifugation moving forward and pellet samples by gravity.

5 Replace with 1 mL fresh fixative (2% paraformaldehyde and 2.5% glutaraldehyde in 0.1 M sodium cacodylate buffer (pH 7.2)) and continue to fix the cells overnight at 4 °C.

Sample staining and embedding

6 Discard fixative and add 1 mL 0.1 M cacodylate buffer for 10 min at room temperature. Repeat this buffer rinsing step two more times.

7 Discard remaining buffer and incubate the sample in freshly made 200 µL reduced osmium solution that contains 2% osmium, 1.5% potassium ferrocyanide in 0.1 M sodium cacodylate buffer, for 1.5 h at room temperature in the dark.

7.1 Caution: do not disturb the cell pellet during all sample processing steps.

8 Wash the sample in 1 mL ddH_2O for 3 min at room temperature. Repeat this wash step four more times.

9 Incubate the sample in freshly prepared 1 mL TCH (thiocarbohydrazide) solution (1%) for 20 min at room temperature.

10 Wash the sample in 1 mL ddH_2O for 3 min at room temperature. Repeat this wash step four more times.

11 Incubate the sample in 200 µL 2% Osmium in ddH2O for 40 min at room temperature.

12 Wash the sample in 1 mL ddH_2O for 3 min at room temperature. Repeat this wash step four more times.

13 IIncubate the sample in 0.5 mL 1% uranyl acetate in aqueous solution, overnight at 4 °C in the dark.

14 Wash the sample in 1 mL ddH_2O for 3 min at room temperature. Repeat this wash step four more times.

15 Incubate samples in 1 mL of En bloc Walton's lead aspartate solution**[1]**, staining for 30 min inside a 60 °C oven.

15.1 Critical step: Walton's lead aspartate solution should be prewarmed at 60 °C for 30 min. Avoid precipitate formation.

16 Wash the sample in 1 mL ddH_2O for 3 min at room temperature. Repeat this wash step four more times.

17 Carry out sample dehydration in a graded ethanol series as follows (1 mL per 1.5 mL tube, or 2 mL per 35 mm dish):
30% ethanol in ddH_2O; 1X for 10 min at 4 °C
50% ethanol in ddH_2O; 1X for 10 min at 4 °C
70% ethanol in ddH_2O; 1X for 10 min at 4 °C
85% ethanol in ddH_2O; 1X for 10 min at 4 °C
95% ethanol in ddH_2O; 1X for 10 min at 4 °C
100% ethanol; 1X for 10 min at 4 °C
100% ethanol; 2X for 10 min at room temperature

18 Carry out resin infiltration of the sample using a graded Durcupan resin series as follows (0.5 mL per 1.5 mL tube, or 1 mL per 35 mm dish):
25% Durcupan in ethanol; 1X for 4 h at room temperature
50% Durcupan in ethanol; 1X for overnight at room temperature
75% Durcupan in ethanol; 1X for 2 h at room temperature
100% Durcupan; 1X for 2 h at room temperature
100% Durcupan; 2X for 1 h at room temperature

18.1 Note: For cell pellet sample embedding, a hard formulation of EMbed **[2]** can replace Durcupan, which is easier for sectioning with an ultramicrotome. However, EMbed **[2]** will dissolve the cell culture dish, therefore it is not appropriate for an *en face* CLEM project.

19 For sample embedding of *en face* samples, tilt the dish at a 45 ° angle in order to discard as much leftover resin from the dish as possible. Add a thin layer of 100% Durcupan resin (~100 µl) directly onto the glass coverslip. For cell pellet samples, there are two options when sample embedding: 1) use a wood applicator stick to transfer the sample to an embedding mold, or 2) directly embed the sample inside the tube if the cell pellet is very small. With either sample embedding method, add 100% Durcupan to fill the embedding mold or ~200 µL to the tube if directly embedding the sample inside the tube. Place embedded samples in a 60 °C oven for 48 - 60 h to allow resin-infiltrated samples to polymerize and harden.

19.1 Critical step: 1) for *en face* samples, be sure to add a requisite volume of 100% Durcupan (one or two drops) that will cover just the glass coverslip and not the surrounding areas of the dish and 2) for cell pellet samples in which the sample is very small, sample embedding directly in the tube is recommended.

19.2 Note: Annotate samples clearly to prevent any confusion downstream when working with multiple samples. For *en face* samples, write down a sample number on a piece of paper and place it on the plastic area of the dish, so that any leftover resin will stick the number tightly to the dish. For cell pellet samples, put a sample number into an embedding mold, preventing overlap with the sample; alternatively, stick it to the inner wall of the tube.

Sample trimming and mounting: cell pellet samples

20 Bend the sample embedding mold, or cut open a tube using a razor blade to take out a polymerized sample block.

21 Lock the sample block on the specimen holder of the microtome, then trim the sample block under the microtome. Trim one edge of the sample block containing the cell pellet using a razor blade to expose the cell sample.

22 Place the sample block in the specimen arc of the ultramicrotome and section the exposed sample surface using a Diatome Histo Diamond knife to create the blockface. The knife holder and specimen arc angles should be set to zero.

23 Take the sample block out of the specimen arc. Using a razor blade, trim the remaining edges of the sample block into a pillar.

23.1 Critical step: While trimming the sample block edges, ensure that: 1) the excess resin is trimmed away on the remaining edges in order to expose the sample, 2) each side of the pillar is of an equal dimension relative to the block face and 3) Typically, samples are trimmed into a pyramid shaped block that contains no regions of empty resin i.e. some of the sample is exposed on every side **[3–5]**. Samples can also be trimmed to a cube/ cuboid shape, ~0.5 mm x 0.5 mm and of a similar height.

24 Place the sample block under a stereomicroscope and cut away the newly created 3View sample blockface pillar with a razor blade.

24.1 Note: Laying the block flat and protecting the pillar with scotch tape before cutting will help to ensure that the pillar is not lost. Doing so on a piece of white paper will contrast the osmium-darkened sample, making the pillar easier to see.

25 Glue the pillar to a 3View SEM pin stub (EMS Cat# 75959-02) using silver conductive epoxy.

25.1 Critical step: While gluing the sample, ensure that 1) the sample block face created using a Diatome Histo Diamond knife is glued directly to the surface of the 3View SEM pin stub, with the side cut away from the sample block facing up and 2) each side of the pillar is also covered by silver epoxy.

26 Place the 3View pin stub in a 60 °C oven overnight to polymerize the epoxy.

27 Place the pin stub in the specimen arc of the ultramicrotome and section the exposed sample surface using a Diatome Histo Diamond knife, where both the knife holder and specimen arc are set to angles of zero.

28 Remove the pin stub from the ultramicrotome and trim away any excess epoxy from the sides of the pillar.

Sample trimming and mounting: *en face* samples

29 Freeze the dish with the resin embedded sample in liquid nitrogen to carefully separate the glass coverslip from the polymerized cell monolayer. Repeat these freeze thaw cycles if necessary.

29.1 Critical step: Immediately after freezing, use a very sharp razor blade to separate the perimeter of the circular coverslip from the dish, by lodging the razor edge between the perimeter of the coverslip and dish while rotating the dish.

30 Identify the region of interest (ROI) and its respective, letter-number location square within the location-based grid on the sample side of the polymerized monolayer (Figure 1B).

31 On the sample side mark off a square around the letter-number location containing the ROI, using a razor blade (Figure 1C).

31.1 Critical step: Take care not to damage the surface of the monolayer-ROI during this and all subsequent steps, as it is no longer protected by the glass coverslip; simply touching it may destroy the sample and/or its respective location-specific letter/number.

32 Turn over the polymerized sample monolayer and mark the location of the ROI with an “X” on the non-sample side of the resin; use the square marked by a razor on the sample side as a reference in doing this (Figure 1C).

33 Using a stereomicroscope, confirm that the “X” on the non-sample side aligns with the ROI on the sample side (Figure 1C).

34 Carefully place a 1 x 2 mm slot grid over the ROI on the sample side. Ensure that the ROI is centered within the slot. Using a razor blade, place marks at the sides of the slot grid so that

the marks are parallel to the 2 mm side of the slot. These marks, in combination with the marks made in step 31, should create a rectangle equal to the diameter of the slot grid (Figure 1C).

35 Using a razor separate this rectangle, one side at a time, from the larger polymerized monolayer (Figure 1C).

35.1 Critical step: Confirm that the non-sample side of the rectangular resin piece contains the centered "X" from step 30 (Figure 1C).

36 Set the rectangular resin piece aside, either in a microcentrifuge tube or a small gelatin capsule, so as not to lose the sample during subsequent steps.

37 Using a conductive silver epoxy, glue two slot grids one on top of the other to the top of a 3View SEM pin stub (EMS Cat# 75959-03) (Figure 1C).

37.1 Note: Make sure that the slots of both slot grids are aligned

38 Place the 3View SEM slot grid-pin stub into a 60°C oven overnight to polymerize the epoxy.

38.1 Note: Once dried, remove the now polymerized, excess silver epoxy from the interior of the superimposed slots using the edge of a razor or scalpel.

39 Glue the ends of the rectangular resin piece to the broader parts of the slot grid, sample side down, using the same conductive epoxy.

39.1 Critical step: Ensure that the ROI target is centered in the slot of the slot grid and that the "X" marked on the non-sample side in Step 30 faces up. Be very careful to avoid getting epoxy on the ROI itself by placing a piece of tape on the sample side.

40 Allow the glue between the 3View SEM-slot grid pin stub and the rectangular resin piece to fully polymerize. Leave samples in a 60 °C oven overnight.

41 Place the 3View SEM slot grid-pin stub into the specimen arc of the ultramicrotome, taking care to ensure that both the specimen arc and knife holder are at zero degrees.

42 Using a Diatome Histo Diamond knife, section the now exposed non-sample side, at 500 nm section thickness and speed 1 mm/second. Continue to section until most of the resin from the non-sample side is removed and the rectangle is as thin as possible.

42.1 Critical step: Thinning the non-sample side of the rectangular resin piece not only removes excess resin in order to increase the conductivity of the sample, but also ensures that the sample is parallel to the 3View SEM pin stub surface.

43 Using a sharp razor, very carefully remove the thinned resin rectangle from the 3View SEM slot grid-pin stub.

44 Under a stereomicroscope, cut the ends of the rectangle away so that the resin piece becomes a square with the ROI centered.

45 Glue this final square resin piece to the 3View SEM pin stub (EMS Cat# 75959-02), sample side up, using a conductive silver epoxy (Figure 1C).

46 Place the 3View pin stub in a 60 °C oven overnight, to polymerize the epoxy.

46.1 Critical step: Sample on 3View pin stub can be baked in a 100 °C oven for 1 h before sputter coating to increase the stability of the sample during SBF-SEM cutting.

47 Load the sample pin stub into a sputter coater. A layer of gold, 15 nm thick is sputtered onto the sample block faces. This improves conductivity and the reflective surface aids in the manual sample approach detailed in step 53.

Sample loading

48 Slot and secure the sample pin stub into the 3View microtome chuck by tightening a set screw. The sample is first rotated inside of the chuck such that the leading face of the block is rotated 90 ° clockwise from the set screw. This is done with the assistance of a stereo microscope.

49 Check that the sample is loaded and secured into the microtome. Shift the diamond knife laterally and adjust the cutting window position to center over the span of the block face. Using a stereo microscope and the reflection of the knife edge on the block face, manually approach the sample to the cutting plane.

49.1 Critical step: Care should be taken to avoid contact between the block face and the knife edge, as this will leave an imprint several microns deep. A practiced user should get the block face 5-10 μm away from the cutting plane.

49.2 Note: Leave the sample inside SEM chamber overnight before imaging to allow for a more thorough off-gassing and temperature equalization of the hardware and sample.

Sample imaging

50 Navigate to an area of interest on the sample block face by using the stage controls in DigitalMicrograph software (Gatan).

51 Perform coarse cuts at 100 nm slice thickness to approach the sample blockface to the ultramicrotome knife. Acquire low magnification reference scans after every other coarse cut to confirm that the sample block is in the cutting plane of the knife.

52 For cell pellet embedded samples, acquire a low resolution scan that captures the sample blockface. Proceed directly to step 55.

53 For *en face* embedded samples, acquire an image with the secondary electron detector. This provides a clearer view of the sample blockface surface and makes visible the debossed grid numbers and borders.

54 Use low magnification images from light microscopy and the secondary electron detector to correlate the position of the cell of interest on the block face.

55 Create and position an imaging tile (or tile-set) over the cell of interest.

56 Set the chamber pressure, aperture size and electron beam settings. Refer to parameters listed in table1.

57 Using the secondary electron detector, activate Beam Wobble in SmartSEM (Carl Zeiss GmbH). Adjust the aperture X and Y position to center and align the mid-lens aperture. When the aperture is centered, the image will show no side-to-side movement and Beam Wobble can be deactivated.

58 In Gatan Digital Micrograph, edit the image tags in the Global Info settings to assign any metadata that is not automatically generated. These tags may include sample name, lab or investigator name, sample block number (if applicable), embedding resin used, aperture size and focal charge compensation (FCC). Imaging parameters such as slice thickness, pixel size, dwell time and acceleration voltage are automatically embedded in the metadata.

59 Assign a storage directory for the saved dataset. Each scan is saved as an individual file in the native Gatan Digital Micrograph '.dm3' file format. Multiple regions of interest are saved into their respective folders (ROI_00, ROI_01, etc.).

60 Begin the dataset collection. For multiple regions of interest, the software will ask to confirm the positioning of the scans. The software will also ask the user to assign a region as a "monitor stack", which allows for the storage of several sequential scans in RAM for quick viewing. This is used to monitor cutting quality, sample charge rate and to perform any focus corrections during data collection.

61 Adjust focus and stigmation as the first image is being acquired.

61.1 Critical step: Be sure to monitor the first scans to assess whether there are any sample charge artifacts in the image. FCC pressure can be increased to compensate for sample charge or reduced to improve image contrast if the sample is sufficiently conductive.

62 Monitor imaging periodically to adjust any focus drift that may occur during acquisition.

62.1

To manually focus the image, set the 3View collection to a Pause state. Using a continuous live image of the block-face, manually navigate to an adjacent area that does not overlap with the region of interest being imaged. Adjust the focus and stigmation manually. Fast Fourier Transform analysis of the images is available, but may provide limited utility due to the relatively low signal-to-noise in the live images. Once the focus has been adjusted, set the 3View collection to Resume. For Multi-ROI imaging mode, the stage will automatically navigate to the next ROI in the image series. For a single ROI acquisition, first manually input the stage coordinates of the collection area and then set the 3View collection to Resume.

Image processing

63 Step 63: Import the image stack into Fiji for image processing. Processing steps can include applying a gaussian blur filter to smooth the image, reducing any noise and correcting any changes in intensity across the image stack using bleach correction plugin. Image stacks can also be aligned in Fiji using the image registration plugin. Export the image stack as 16-bit tiff images and analyze them using a segmentation software of your choosing.

Protocol references

1. Walton J. Lead asparate, an en bloc contrast stain particularly useful for ultrastructural enzymology. J Histochem Cytochem. 1979;27: 1337–1342.
2. Luft JH. Improvements in epoxy resin embedding methods. J Biophys Biochem Cytol. 1961;9: 409–414.
3. Borghgraef P, Kremer A, De Bruyne M, Guérin CJ, Lippens S. Chapter 2 - Resin comparison for serial block face scanning volume electron microscopy. In: Narayan K, Collinson L, Verkade P, editors. Methods in Cell Biology. Academic Press; 2023. pp. 33–54.
4. Titze B, Genoud C. Volume scanning electron microscopy for imaging biological ultrastructure. Biol Cell. 2016;108: 307–323.
5. Guérin CJ, Kremer A, Borghgraef P, Lippens S. Targeted Studies Using Serial Block Face and Focused Ion Beam Scan Electron Microscopy. J Vis Exp. 2019. doi:10.3791/59480
6. Antao NV, Lam C, Davydov A, Riggi M, Sall J, Petzold C, et al. 3D reconstructions of parasite development and the intracellular niche of the microsporidian pathogen Encephalitozoon intestinalis. Nat Commun. 2023;14: 7662.
